# The Quantity‐Competence Presumption: Children Use Informativeness to Calibrate Trust and Infer Speakers' Knowledge

**DOI:** 10.1111/desc.70276

**Published:** 2026-08-25

**Authors:** Cyann Bernard, Adeline Depierreux, Olivier Mascaro

**Affiliations:** ^1^ Integrative Neuroscience and Cognition Center Université Paris Cité, CNRS Paris France

**Keywords:** cognitive development, pragmatics, social learning, theory of mind, trust

## Abstract

Choosing whom to learn from is critical for human learners. A well‐established strategy to do so is to track informants' past accuracy. Yet this requires both a track record and independent knowledge of the truth—neither of which is available when encountering a new informant in an unfamiliar domain. This paper proposes that the amount of information an individual communicates can serve as a proxy signal for their trustworthiness, and tests this hypothesis in children. Study 1 (*N* = 225, ages 4–11) pitted an informant making necessarily accurate but uninformative tautological claims (e.g., “a dibleur is a dibleur”) against one making informative claims of unknown accuracy (e.g., “a dibleur is a bird”). Children as young as four preferred to seek and endorse information from the more informative speaker—even though her accuracy had never been established. This preference was bounded: when the non‐tautological speaker's claims were clearly inaccurate, children shifted their trust to the uninformative but accurate speaker. Study 2 (*N* = 159, ages 4–11) tested the cognitive bases of reliance on informative individuals. It assessed whether children apply the quantity‐competence presumption: all else equal, a more informative individual implies she knows more. Children attributed greater knowledge to more informative speakers across four conditions varying in the degree and nature of the informativeness contrast. Together, these findings reveal that children treat informativeness as a graded signal of reliability, using it to bootstrap trust in unfamiliar informants when other epistemic tools are unavailable.

## Summary

- Children take calculated epistemic risks.
- They trust sources whose claims are informative but whose accuracy is not known over certainly accurate individuals who state the obvious.
- Inferences about minds support trust in informative individuals. Children treat the amount of information speakers provide as a cue of their knowledgeability.
- These findings reveal a bootstrapping mechanism—the quantity‐competence presumption—enabling children to calibrate trust in unfamiliar informants without any accuracy track record.

## 1 Introduction

### 1.1 A Bootstrapping Problem in Deciding Whom to Trust

Communication is instrumental to human learning, making it critical to choose whom to learn from (Harris 2012; Mercier 2020; Mills 2013; Sperber et al. 2010). Learners must avoid deception or error (Clément et al. 2004; Koenig et al. 2004; Mascaro and Sperber 2009; Vanderbilt et al. 2011). Moreover, even in the absence of active deception or blatant mistakes, learners face opportunity costs: settling for a sub‐optimal informant when a better one was available means missing on opportunities to learn.

A natural response to this challenge is to track the past accuracy of informants—whether what they communicated was actually the case—and use this history to determine whom to trust (Clément et al. 2004; Hermes, Behne, Bich, et al. 2018; Hermes, Behne, Rakoczy, et al. 2018; Heyman 2008; Koenig and Harris 2007; Mascaro and Morin 2015; Shafto et al. 2012; Sobel and Kushnir 2013). Developmental research shows that children do exactly this: from early in development, they preferentially learn from informants who have demonstrated accuracy over those who have made errors (Begus and Southgate 2012; Chow et al. 2008; Clément et al. 2004; Corriveau and Harris 2009; Dautriche et al. 2021; Koenig et al. 2004; Koenig and Woodward 2010; Ronfard and Lane 2018, 2019; for meta‐analyses see Sobel and Finiasz 2020; Tong et al. 2020). Yet this strategy is not without limits. It requires both a track record and independent knowledge of the truth—neither of which is available when a learner first encounters a new informant in an unfamiliar domain. Children, who are perpetually newcomers to some area of knowledge, face this challenge constantly.

This paper proposes a solution to this bootstrapping problem. We argue that children use the sheer *amount* of information an individual communicates as a proxy signal for their trustworthiness—even without any evidence about the accuracy of their claims. Throughout the paper, we characterize informativeness as the capacity of a statement to reduce uncertainty over a set of hypotheses (Frank and Goodman 2012; Shannon 1948): the more a statement narrows the field of possibilities, the more informative it is. Importantly, informativeness is distinct from accuracy. A field guide declaring “all mushrooms with a red cap are poisonous” is highly informative—she drastically reduces uncertainty about which mushrooms to avoid. She is also, as any serious mycologist will tell you, mistaken. The red‐capped chanterelle is perfectly edible, and rather delicious. Informativeness is no guarantee of accuracy, and vice versa.

### 1.2 Expectations of Informativeness and Their Development

A good informant is one whose contributions are cognitively worthwhile—that is, one whose communications yield cognitive benefits sufficient to justify the effort required to process them (Sperber and Wilson 1986). When the processing costs outweigh the benefits, or when the benefits are negligible, an act of communication fails to be sufficiently cognitively useful. Subsequently, the proponents of relevance theory have argued that communicating carries an implicit commitment. By the very act of signaling their intention to communicate, speakers present their utterances as relevant—that is, as having a sufficiently good, if not optimal, cost/benefit ratio, making it worth the audience's attention and processing effort (Sperber and Wilson 1986; Wilson and Sperber 2012). Sufficient informativeness, in this framework, is not an independent expectation that listeners appeal to when interpreting communicated contents; it is more simply one of the properties that can contribute to making an utterance relevant. Other frameworks—Grice's maxim of quantity (Grice 1989), Levinson's Q‐Principle (Levinson 1998), and the Rational Speech Act framework (Frank and Goodman 2012; Goodman and Frank 2016)—reach convergent, if differently grounded, conclusions. Their empirical predictions converge on this point: listeners routinely assume that speakers are sufficiently informative, and interpret what speakers intend to convey accordingly.

These predictions receive empirical support by a very young age. On the comprehension side, children as young as two‐and‐a‐half already interpret the communicative acts of others by assuming sufficient informativeness (Aguirre et al. 2023). Similar processes shape lexical acquisition from around 3 years of age (Bohn et al. 2021; Frank and Goodman 2014), though they appear to be absent in infancy (Aguirre et al. 2025). On the production side, children's non‐verbal communication is tailored to their audience's knowledge state: they are more likely to communicate something unknown to their interlocutor than something already known, both when informing (Liszkowski et al. 2007) and when attempting to persuade (Mascaro et al. 2019). By two‐and‐a‐half, children also adapt their gestures and speech to make their intended meaning clearer for listeners (Abbot‐Smith et al. 2016; Grigoroglou and Papafragou 2019; O'Neill and Topolovec 2001), and by 3 years they preferentially communicate unexpected, informative features when describing pictures (Bannard et al. 2017). This convergence across comprehension and production is telling. It suggests that assuming sufficient informativeness is an early‐emerging feature of children's engagement with communication—shaping both how they interpret others and how they present information themselves.

Children also evaluate negatively speakers who, by being under‐informative, imply something inaccurate. Evidence for this ability comes from work on “scalar implicatures”—for example, inferring that by saying “Bob ate some of the cookies”, one implies he did not eat all of them. Although young children often struggle to spontaneously derive scalar implicatures (Noveck 2001; Papafragou and Musolino 2003), by around 5 years and under supportive conditions, they evaluate under‐informative speakers more negatively than fully informative ones (Foppolo et al. 2012; Katsos and Bishop 2011; Skordos and Papafragou 2016). For instance, Katsos and Bishop (2011, Exp. 2) found that 5–6‐year‐olds gave lower rewards to a speaker who said that a mouse collected “some carrots” when it actually collected all of them, compared to a fully informative speaker who said that it collected “all carrots”. Children also rate negatively teachers who commit “sins of omission”—omitting relevant information during demonstrations, thereby implying something false. In a series of key studies, Gweon et al. (2014) showed that 6‐ and 7‐year‐olds rated a teacher more negatively when that teacher had demonstrated only one of the functions of a multi‐function toy—implying, misleadingly, that the toy had only one function, when in fact, it had several ones. Even 4‐year‐olds, in supportive conditions, can penalize teachers who mislead by withholding information (Bass et al. 2022; Gweon and Asaba 2018).

Together, these findings establish that informativeness is a dimension along which children evaluate informants. They also reveal an important boundary condition on existing research: in the studies reviewed above, children already knew the relevant ground truth. For instance, in studies by H. Gweon and colleagues, informants communicated about toys whose functions had been previously shown to children (Bass et al. 2022; Gweon et al. 2014; Gweon and Asaba 2018). Thus, children could directly detect the mismatch between what an informant communicated and what was actually the case. In these contexts, evaluating under‐informative informants was a test of children's ability to draw pragmatic inferences—that is, to infer what the speaker implied when being under‐informative. It was not a test of their ability to calibrate trust in the face of genuine uncertainty. Moreover, when children negatively evaluate informants who mislead their audiences by withholding information, there is no tension between evaluations of accuracy and informativeness. In those cases, the least informative individuals are also misleading their audiences by implying something inaccurate, giving two different reasons not to trust them.

### 1.3 The Tension Between Informativeness and Accuracy

This paper addresses a very different and quite difficult situation: when the learner has no independent access to the ground truth. This is a typical situation for learning through communication that children encounter routinely, and it poses the question of deciding whom to trust in its sharpest form.

Just think about what children are asked to accept on a regular school day: that blood is pumped by a muscle the size of a fist that they cannot see working; that the Moon does not actually change shape but only appears to do so; or that one of their distant ancestors was, biologically speaking, a fish. Quite often, such claims do not come with a means for the child to directly assess their accuracy. The child cannot pull back a curtain to check. She cannot conduct an experiment, rely on her own senses, or cross‐check this information with what she already knows, because in each case, what is being told to her falls precisely into the category of things over which a young child has absolutely no independent control. So, she has to take someone's word for it—and the crucial question left to her, whether she asks it consciously or not, is who that “someone” is.

What makes it harder still is that informativeness and accuracy are not merely distinct—they stand in a principled tension. The more informative a claim, the more ways it has to be wrong. Consider two informants asked to tell you something about a novel entity—call it a “*dibleur*”. The first plays it safe. “*A dibleur*,” she announces, “*is a dibleur*”. She is, of course, perfectly correct; statements of the form *A is A* are necessarily true. But they are also perfectly uninformative. The second informant says: “*A dibleur is a bird*”. Suddenly, things get interesting—and risky. This claim could be inaccurate. Maybe *“dibleurs”* are reptiles. Maybe “*dibleur*” is the name of a jazz club. The moment genuine information enters the picture, so does the possibility of error. That is not a bug; it is a feature of what information is. To learn something from an utterance, you have to be in a world where that utterance could, in principle, have been inaccurate.

Suppose our second informant goes further: “*A dibleur is a bird with a very long yellow beak*”. More informative still—and now more fragile. Any fact that falsifies the simpler, less informative, claim (“*A dibleur is a bird*”) falsifies this one too. But the reverse does not hold. Discover that dibleurs have black beaks, and the richer claim crumbles while the humbler one survives. Put formally, if claim *B* strictly entails claim *A*, then any state of affairs that falsifies *A* necessarily falsifies *B*—but the reverse does not hold. Thus, the falsification conditions of *B* are a proper superset of those of *A*. In short, gains in informativeness are purchased at the cost of increased vulnerability to error.

This trade‐off creates a genuine tension for the learner, and the first study in this paper investigates how children navigate it. One possibility is that children are epistemically conservative. Based on their preference for accuracy, they might favor informants whose claims are uninformative, but known to be true, over those who are informative, but whose accuracy is not known. In fact, when children are faced with conflicting cues of trustworthiness, past accuracy often trumps other factors, such as familiarity, age, accent, gender or consensus (Bernard et al. 2015; Corriveau et al. 2013; Corriveau and Harris 2009; Jaswal and Neely 2006; Taylor 2013; for exceptions see: Bascandziev and Harris 2016; Williams and Danovitch 2024).

Alternatively, children might accept epistemic risk in order to make room for learning opportunities, preferring to learn from individuals who communicated information whose accuracy is uncertain, rather than from individuals whose accuracy track record is impeccable, but provided no information. Accuracy alone is not sufficient to make communicated information useful—a speaker who only states obvious facts, although perfectly accurate, contributes little to learning. Hence, learning progress based on communication requires some openness to information whose accuracy is uncertain.

Study 1 was designed to judge between these two hypotheses. It builds on previous research on children's evaluation of circular or non‐circular explanations and arguments (Baum et al. 2008; Castelain et al. 2018; Corriveau and Kurkul 2014; Mercier et al. 2014, Mercier et al. 2018; Mills et al. 2017, Mills et al. 2019). Importantly, those past studies focused on explanatory and argumentative quality—namely, whether statements provide reasons accounting for a phenomenon, or in support of a conclusion. By contrast, Study 1 focuses on children's sensitivity to the speakers’ informativeness per se. For this reason, it deliberately uses novel labels and non‐existing entities, and no argument or causal explanation.

In Study 1's test condition, children encounter two informants. One makes tautological claims that are necessarily accurate but uninformative—for example, “*a dibleur is a dibleur*”. The claims of the second informant are non‐tautological, for example, “*a dibleur is a bird*”—informative, but of unknown accuracy, since the child knows nothing about dibleurs. If children tolerate epistemic risks to support learning progress, they should rely more on the non‐tautological, informative individual than on the tautological one. Conversely, if children are epistemically conservative, valuing certain accuracy above all, they should rely more on the tautological informant—who demonstrates accuracy—than on the non‐tautological one. The control condition tests the boundary of the effects observed in the test condition by replacing unknown accuracy with obvious inaccuracy. In that case, the non‐tautological informant now makes clearly inaccurate claims—thus providing a clear reason not to trust her.

### 1.4 Pre‐registration, Materials, and Data Sharing

The studies reported in this article were pre‐registered. Pre‐registrations, data, stimuli samples, and analysis scripts of all Studies are accessible in an open repository (Study 1: https://osf.io/cfm5z/?view_only=2b80af72d73045c4bd3b5bb6971e8ed2; Study 2: https://osf.io/g5u79/?view_only=ddbd284bd1e348cdada3a7dd3ef90206).

### 1.5 Ethical Considerations

The research reported in this manuscript followed the guidelines of the Declaration of Helsinki and was approved by an independent ethics committee (CER U‐Paris Cité, IRB: 00012023‐102). The parents of all participants gave their informed written consent before their inclusion in any of the studies.

## 2 Study 1

### 2.1 Method

#### 2.1.1 Participants

Two hundred and twenty‐five children aged from 3 years and 11 months to 11 years and 4 months participated in Study 1. They were enrolled in a test condition (*n* = 115, 63 girls; *M_age_* = 7 years and 3 months, *SD_age_* = 2 years) or a control condition (*n* = 110, 60 girls, *M_age_* = 7 years and 2 months, *SD_age_* = 2 years and 1 month). Five additional participants were tested but excluded for the following reasons: technical failure (1), providing non‐sensical responses unrelated to the task (2), refusing to interact with the experimenter (1) or to perform the task (1). Sample sizes were set by conducting an a priori power analyses with G*power (v.3.1; Faul et al. 2007), assuming a medium effect size (*d* = 0.5). Based on these analyses, we aimed for a minimum sample size of 90 participants per condition in Study 1, which is sufficient to achieve a power higher than 0.80 (*α* = 0.05) for comparing means across two independent groups with a two‐tailed Mann–Whitney *U*‐test. Following our recruitment efforts, more participants met our criteria in the schools contacted for the study and were included beyond the minimum of 90 participants. For all studies, the participants were recruited in a large metropolitan area (Paris and its surroundings). Data on ethnicity were not collected, as this is prohibited in France. Additional details about demographic information and recruitment procedures are in the Supporting Information.

#### 2.1.2 Apparatus

Children were tested individually in a quiet room. They sat at a table next to the experimenter, facing a laptop where the stimuli were displayed (using PowerPoint). A camera (temporal resolution = 25 frames per second) recorded the participants’ behavior.

#### 2.1.3 Test Condition

The test condition of Study 1 was divided into three consecutive phases: an initial phase, an explicit judgement phase, and a generalization phase.

##### 2.1.3.1 Initial Phase

After a brief warm‐up (detailed in the Supporting Information), participants were enrolled in an initial phase with four trials. During each of them, children had to discover the meaning of a novel pseudo‐word (*dibleur*, *granée*, *mouval* or *ludine*; a different pseudo‐word was used in each trial). First, children were asked if they knew the meaning of a novel pseudo‐word (e.g., *dibleur*). If they claimed to know, the experimenter corrected them.

**FIGURE 1:**
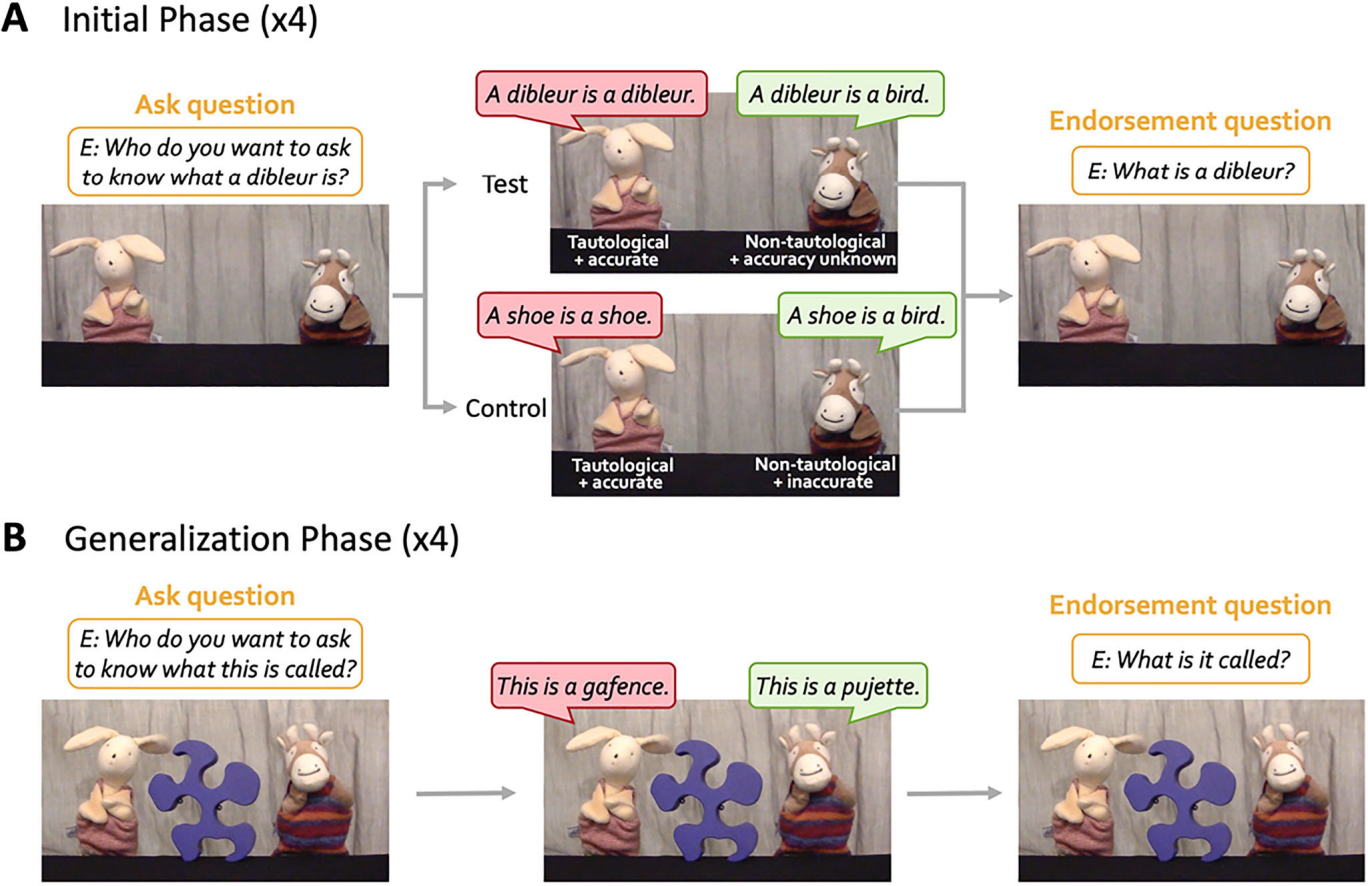
Trial sequence for the initial and generalization phases of study per condition. *Note*: Panel A depicts the sequence of events unfolding during the four trials of the initial phase in the test and control conditions of Study 1. First, participants were asked to choose one character to tell them about the meaning of a novel label. Next, what characters said varied across conditions. In the test condition, the tautological character made an uninformative but accurate claim, while the non‐tautological character made an informative claim whose accuracy was not known. In the control condition, the tautological character still made an uninformative but accurate claim, while the non‐tautological character made a claim that, whilst not tautological, was inaccurate. At the end of each trial of the initial phase, participants were asked to endorse the claim of one of the two informants. Panel B depicts the sequence of events unfolding during the four trials of the generalization phase. In both conditions, children had to choose from which informant to ask and endorse information when learning the name of an unknown object. The text, arrows, and speech bubbles shown in the figure serve illustration purposes. They did not appear in the videos shown to the participants (E = experimenter).

Next, the experimenter posed an “ask” question: *“Let's ask the rabbit and the cow what a [dibleur] is. Who do you want to ask what a [dibleur] is?”*. Subsequently, the participants watched a video in which each of the two characters uttered something about the novel label. One of the puppets—henceforth, the tautological character—uttered a tautology that was uninformative but accurate: *“A [dibleur] is a [dibleur].”* The second puppet—henceforth, the non‐tautological character—made a claim that was informative, but whose accuracy was not known, for example: *“A [dibleur] is a [bird]”* (Figure 1A). The non‐tautological character's utterance used an existing word familiar to children to explain what the pseudo‐word referred to (i.e., *bird*, *fruit*, *cake*, and *flower*; with a different familiar word being used in each trial, see Table S1). For each participant, each animal puppet always acted either as the tautological character, or as the non‐tautological character. Note that in the videos used in the initial phase, there were no visible unfamiliar objects that could serve as referents for novel labels—only the two characters were present.

Once the two characters had spoken, the experimenter asked an “endorse” question: “The rabbit says that a [dibleur] is a [dibleur], and the cow says that the [dibleur] is a [bird]. According to you, what is a [dibleur]?”. If the child did not respond, the experimenter added: “Do you think that a [dibleur] is a [dibleur] like the rabbit says or that a [dibleur] is a [bird] like the cow says?”. Once the child had responded, the experimenter moved on to the next trial, till the completion of the initial phase's four trials.

##### 2.1.3.2 Explicit Judgement Phase

Once the initial phase was over, the experimenter collected the children's explicit judgement by asking them: “Was the rabbit good or not so good at explaining things? And was the cow good or not so good at explaining things? Which animal was the best at explaining things?” (explicit judgement question). Afterwards, the experimenter introduced the generalization phase by saying: “Now we are going to look at some objects and we will have to say what they are, ok?”

##### 2.1.3.3 Generalization Phase

The generalization phase was modelled after Koenig et al. (2004), and Koenig and Harris (2005). It included four trials. At the start of each of them, participants saw a picture of the two characters with an unknown object between them, at the center of the screen. First, the experimenter pointed towards the unfamiliar object, and asked: *“Do you know what this is called?”* Just like in the initial phase, participants claiming to know the answer were corrected. Next, the experimenter posed an “ask” question: *“Let's ask the rabbit and the cow. Who do you want to ask what it's called?”*. Subsequently, the participants watched a video in which each character gave a different name to the unfamiliar object. For example, the first character said: *“This is a [pseudo‐word, e.g., gafence]”* and the second one said: *“This is a [pujette]”* (Figure 1B). Different names and unfamiliar objects were used in each trial of the generalization block (see Table S2). Next, the experimenter asked an “endorse” question: *“The rabbit says it's a [gafence], and the cow says it's a [pujette]. According to you, what is it called?”*. Once the child had responded, the experimenter proceeded with the next trial, till completion of the four trials of the generalization phase.

#### 2.1.4 Control Condition

Study 1's control condition assessed the limits of children's reduced reliance on uninformative individuals. In Study 1's control condition, the procedure was the same as in the test condition, with one exception. In the videos used during the initial phase of the control condition, pseudo‐words were replaced with real familiar words (i.e., *car*, *shoe*, *book*, and *dog*, Table S1). Consequently, the non‐tautological character became inaccurate (saying e.g. *“A [bird] is a [shoe]”*) while the tautological character remained uninformative and accurate (saying e.g. *“A [shoe] is a [shoe]”*, Figure 1B).

#### 2.1.5 Counterbalanced Factors

The factors counterbalanced for Study 1 are detailed in the Supporting Information.

#### 2.1.6 Coding

For each ask and explicit judgement question, participants received a score of 1 if they chose the non‐tautological character, and 0 otherwise. Similarly, for each endorse question, participants received a score of 1 if they endorsed the answer from the non‐tautological character, and 0 otherwise. Data from the ask question of the first two trials of the initial phase were not included in analyses (to ensure that participants would have enough evidence about the characters’ past behaviors when choosing which one to ask for help).

#### 2.1.7 Data Analysis

For all Studies, statistical tests were two‐tailed and conducted with R version 4.1.3 (R Foundation for Statistical Computing, Vienna, Austria), using the following packages: lme4 v.1.1‐36, and rstatix v.0.7.2. Data curation and visualization was achieved using: broom v.1.0.7, knitr v.1.49, cowplot v.1.1.3, ggeffects v.2.2.0, ggplot2 v.3.5.1, ggpubr v.0.6.0., officer v.0.6.8, optimx v.2024‐12.2, patchwork v.1.3.0, plyr, v.1.8.9, purrr v.1.0.4, tidyverse v.2.0.0. Analyses were pre‐registered, with a few minor changes listed in the Supporting Information.

### 2.2 Results

Answers to the ask and endorse questions were analyzed separately, as they differed significantly from each other, in each phase and for each condition of Study 1 (all *W_+_* s > 625, all *p*s < 0.004, all *r*s > 0.26, Wilcoxon tests for matched pairs).

**FIGURE 2:**
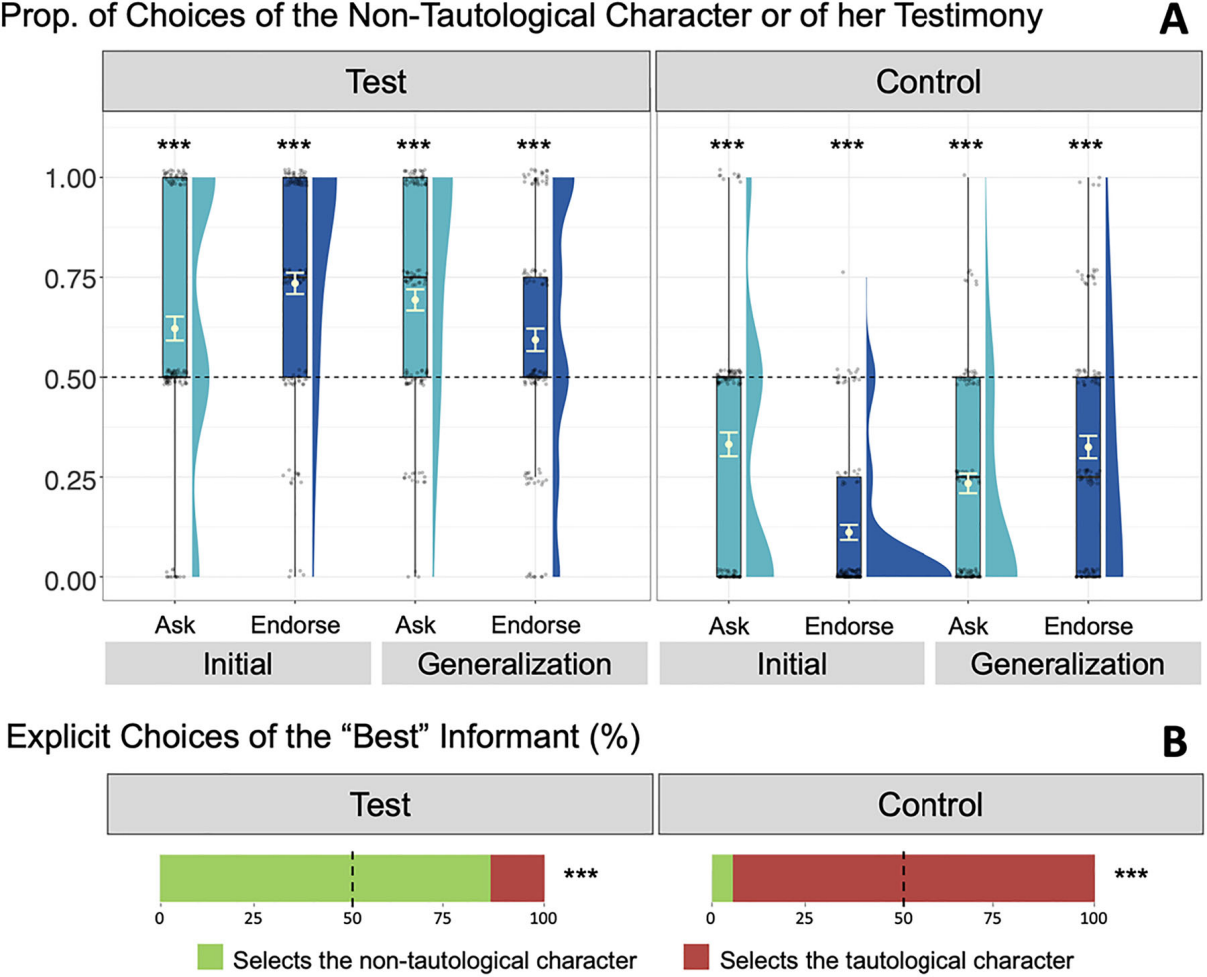
Children's choices in Study 1 per condition, question type and phase. *Note*: Panel A represents the proportion of trials for which participants asked the non‐tautological character, or endorsed her answer, per question type, phase and condition. Yellow dots and error bars represent means and standard errors. Box plots represent data quartiles. Grey dots represent individual data points (with a slight jitter). Colored areas represent data density. Comparisons against chance level (0.5) by one‐sample Wilcoxon signed rank tests. Panel B represents the proportion of answers for the explicit judgement question per condition. Comparisons against the proportion predicted by chance (0.5) by two‐choice binomial tests. The dotted black lines represent the average value predicted by chance (0.5 for Panel A, and 50 for Panel B). Significance codes: *p* < 0.05 = *; *p* < 0.01 = **; *p* < 0.001 = ***.

In the test condition, Study 1's participants asked and endorsed information from the non‐tautological character significantly more often than predicted by chance, during both the initial and generalization phases (all *p*s < 0.001, all *r*s > 0.29, one‐sample Wilcoxon tests, see Figure 2A and detailed statistics in Table S3). Similarly, when asked the explicit judgement question, participants enrolled in the test condition selected the non‐tautological character as the best at explaining things more often than predicted by chance (Figure 2B, 98 participants out of 114, *p* <.001, *g* = 0.36 [0.28, 0.42], binomial test).

Participants enrolled in Study 1's control condition showed the exact opposite pattern to that observed in the test condition. They asked and endorsed information from the non‐tautological character significantly less often than predicted by chance (all *p*s < 0.001, all *r*s > 0.48, one‐sample Wilcoxon tests, Figure 2A, Table S3). They also did so significantly less often than participants in the test condition (all *p*s < 0.001, all *r*s > 0.40, Mann–Whitney *U* test, Table S4).

To look further into effects of age and condition, we also ran Generalized Linear Mixed Models—GLMMs (binomial distribution, logit link). For each of these analyses, we built (1) a null model including the fixed effect of gender, as well as the random effect of participants, and (2) a full model adding the fixed effects of condition (test vs. control) and age (in months, standardized) to the null model. Interactions between age and condition were non‐significant and thus excluded, as pre‐registered.

**FIGURE 3:**
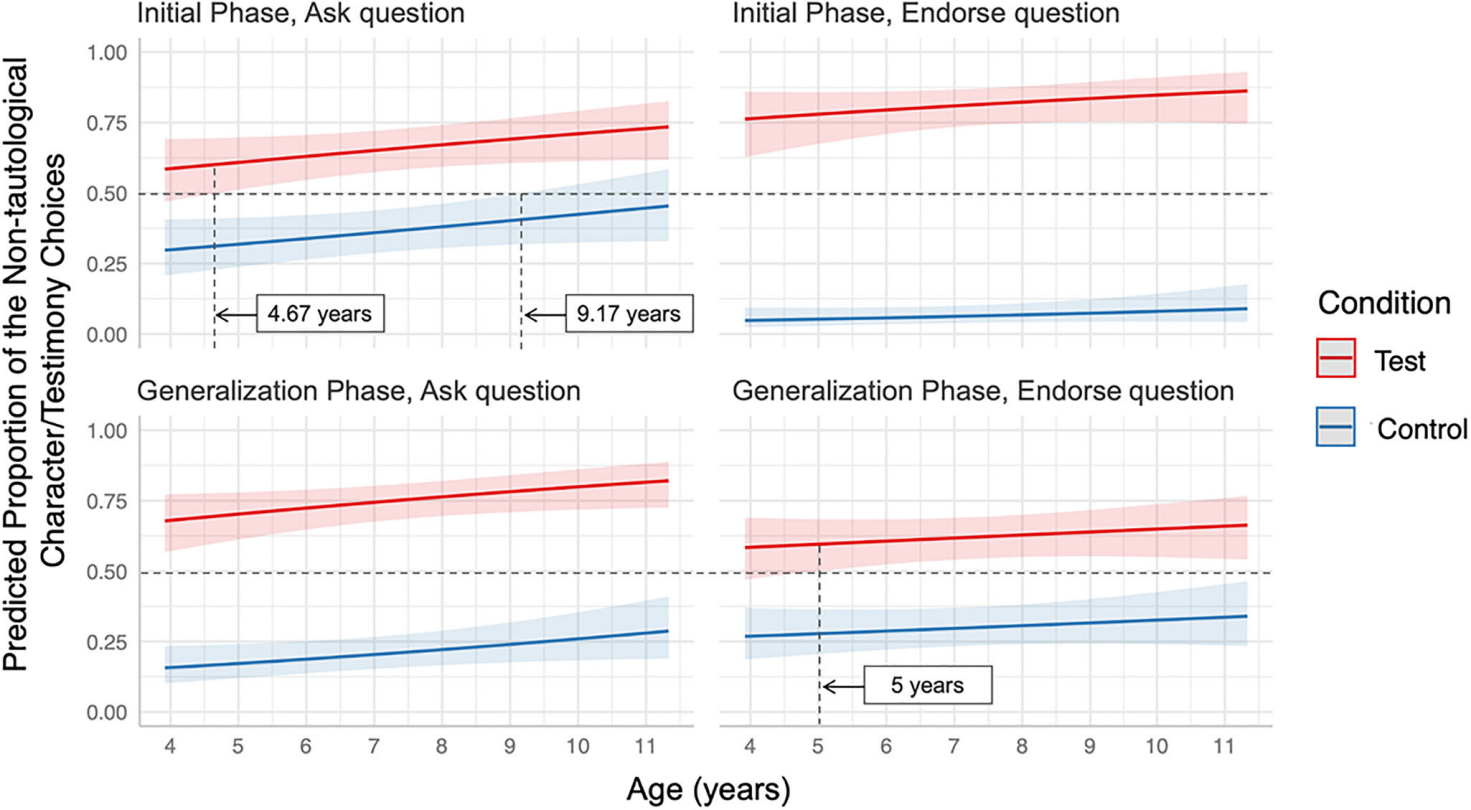
Predicted proportions of choices in Study 1 per condition, question type, and phase. *Note*: Predicted proportion of choices of the non‐tautological character (ask question), or of her testimony (endorse question). Shaded areas represent 95% confidence intervals. Vertical black dotted lines represent the age at which confidence interval did not include chance (0.5), represented by horizontal dotted black lines. Predictions reported on the figure cover the full age range of participants in Study 1 (from 47 to 136 months). For ease of reading, standardized age values used to build the models were back‐transformed to their original values.

These series of GLMMs were run four times, each time with answers on one of the questions (ask or endorse) during one of the phases (initial or generalization phases) as the repeated dependent measure (coded as 1 for choices of the non‐tautological character or of her testimony, and 0 otherwise). In all four cases, the full models had a better fit than the null models (all *χ* ^2^(2) > 41.10, all *p*s < 0.001, Likelihood Ratio Tests). In all four full models, the fixed effect of condition was statistically significant (all *ps* < 0.001; initial phase, ask question: *β* = –1.20, *SE* = 0.20, *z* = –6.03; initial phase, endorse question: *β* = –4.15, *SE* = 0.38, *z* = –10.89; generalization phase, ask question: *β* = –2.43, *SE* = 0.24, *z* = –10.13; generalization phase, endorse question: *β* = –1.34, *SE* = 0.21, *z* = –6.51). The full models revealed no other statistically significant effect. As Figure 3 shows, children in the test condition preferred the non‐tautological character above chance by age 4.67 in the initial phase and across all ages in the generalization phase. They endorsed her testimony above chance across all ages in the initial phase and by age 5 in the generalization phase. Thus, by age 4–5, children consistently preferred informative over uninformative informants.

In Study 1's control condition, children relied more on the tautological character (uninformative but accurate) than on the non‐tautological inaccurate character across all ages, phases, and question types, with one exception: in the initial phase, this preference was no longer significant by age 9.16 years (110 months). The effect of study was significant across the entire age sample (with no overlap between the 95%CI for the two Studies), for all question types, and test phases. Thus, by age 4, children were more likely to ask help from the non‐tautological character and to endorse her testimony when her accuracy was not known (in the test condition) than when she was or had been clearly inaccurate (in the control condition). Additional pre‐registered analyses—assessing learning effects during the initial phase of Study 1 are detailed in the Supporting Information.

### 2.3 Discussion

In Study 1's test condition, the children had to decide whom to trust to learn about new entities designated by pseudo‐words. In the initial phase, one character made tautological statements that were necessarily true but lacked informational content. The accuracy of this informant was therefore undeniable. However, she provided no learning benefit. The other character, who was not tautological, was informative. Yet, it was impossible to know whether what she said was accurate. Then, in the generalization phase, both characters made contradictory suggestions regarding the names of unfamiliar objects. To decide whom to trust at this stage, the children therefore had to rely on the informants’ past accuracy and informativeness, demonstrated during the initial phase.

We found that children consistently preferred to ask for and endorse information provided by the non‐tautological character—the more informative one—in both the initial and generalization phases. This tendency was detectable on some measures by age four, and consistent across all measures by age five. Previous research had shown that, starting at ages 4 to 5, children negatively evaluate an individual who, by communicating in an under‐informative manner, implies something false (Bass et al. 2022; Gweon et al. 2014; Gweon and Asaba 2018; Katsos and Bishop 2011). We find that even when they cannot know whether what is communicated is accurate, children prefer to learn from individuals who provide informative testimonies that can help expand or modify their stock of representations. Children are therefore willing to take epistemic risks when doing so provides an opportunity to learn something new.

This willingness does, however, have its limits. In the control condition, children had to choose between a tautological informant and a non‐tautological but inaccurate informant. In this case, they preferred the accurate informant, in line with numerous previous findings (Clément et al. 2004; Koenig et al. 2004; Koenig and Harris 2005; Sobel and Finiasz 2020; Tong et al. 2020). Thus, children do not avoid tautological informants at all costs when alternative sources of information are clearly inaccurate. These results highlight one of the functions of otherwise uninformative statements—by communicating something that their audience already knows, informants can demonstrate their reliability.

Study 1 also revealed an unexpected finding. During the initial phase of the control condition, younger participants significantly preferred to ask the accurate informant rather than the inaccurate one. However, this preference was not significant among children older than 9.17 years. This age‐related difference is understandable. During the initial phase of the control condition, participants already knew which familiar objects the characters were talking about (for example, a shoe). Furthermore, neither character provided useful information about these objects (e.g., one claimed that a shoe was a shoe, while the other claimed that a shoe was a bird). The younger children nevertheless seem to have considered the accurate statement to be the most appropriate answer, leading them to prefer asking for information from the tautological but accurate informant. It is plausible that the older participants, on the other hand, more readily recognized that neither statement actually taught them anything: being told “a shoe is a shoe” is no more useful than being told “a shoe is a bird” when one already knows what a shoe is. Thus, when asked to choose a source of information to learn more about one of these familiar objects, the 9‐ to 11‐year‐old children may not have shown any preference simply because, from their perspective, both informants were equally useless. It is important to note, however, that these older children were not insensitive to the accuracy of the information provided by the characters in the control condition of Study 1. During the generalization phase, when they actually needed help identifying unknown objects, they relied more on the informant who had previously been accurate (albeit in a tautological way) than on the one who had previously been inaccurate.

In summary, Study 1 demonstrates that children take calculated epistemic risks. They place greater trust in informative individuals whose accuracy is uncertain than in those who provide statements that are necessarily accurate but uninformative. However, even within the context of our study, children remain sensitive to accuracy: they withdraw their trust in non‐tautological individuals when those individuals are clearly inaccurate.

Study 2 examines the mechanisms underpinning children's preference to learn from more informative individuals, even without any direct evidence about the accuracy of what they communicate. We hypothesize that an individual's level of informativeness is an indicator of their level of knowledge—a detailed map implies the mapmaker knows the territory, a sommelier's precise description conveys deep wine knowledge. We call this the “quantity‐competence presumption”: all else being equal, by providing more information, an individual implies that they know more.

Put yourself in the shoes of a child discovering the word “robin”. To learn more, our amateur ornithologist turns to her cousins Reginald and Cornelia. Reginald simply says “*European robins are birds*”, while Cornelia provides more details, stating “*European robins are birds that can perceive magnetic fields thanks to special molecules in their retinas*”. By giving more information, Cornelia implies that she has sufficient knowledge to do so—one can infer that she is likely to know more about robins than Reginald, even without learning anything more about robins or the informants themselves. A larger amount of information is therefore an indirect indicator of knowledge.

This inference stems from fundamental properties of communication and cognition. It is plausible that speakers convey a presumption regarding the cognitive utility of what they communicate (Sperber and Wilson 1986). Subsequently, making claims that contain more information implies that one has more useful information to convey. Similarly, according to the inferential social learning account (Bonawitz et al. 2011; Gweon 2021; Shafto and Goodman 2008), learners assume that teachers select content that maximizes their learning. As a result, a generous sharer who gives little must have little to give. Second, the ability to provide more information serves as indirect evidence of actual reliability: it is relatively easy to construct a detailed, plausible, and consistent account in a field one is familiar with, but far less so in a domain one is not. Third, the more information an individual's testimony contains, the greater the risk of refutation. For instance, tautological informants take no risk: they cannot be proven wrong. More generally, and as noted above, any evidence that refutes a less informative statement—“*a dibleur is a bird*”—also refutes the more detailed statement such as “*a dibleur is a bird with a very long yellow beak*” (but the reverse is not true). Thus, a speaker providing more information makes it easier for external observers to test and evaluate their knowledge. By contrast, being less precise may signal uncertainty, or constitute a form of hedging against potential refutation.

The quantity‐competence presumption is particularly useful when other epistemic tools are lacking—for example, in areas where one's own knowledge is still limited, making it hard to assess the accuracy of informants. Study 2 tests whether children indeed infer an individual's level of knowledge based on the informativeness of their claims. To test the fine‐grained nature of children's inference, they were presented with individuals making claims ranging from non‐informative tautologies (*“a dibleur is a dibleur”*), to simple informative statements (*“a dibleur is a bird”*), and to even more informative ones (*“a dibleur is a bird with a very long yellow beak”*).

## 3 Study 2

### 3.1 Method

#### 3.1.1 Participants

One hundred and fifty‐nine children aged from 4 years and 1 months to 10 years and 11 months participated in Study 2 (75 girls, *M_age_* = 7 years and 9 months, *SD_age_* = 1 year and 11 months). Four additional participants were tested but excluded because they did not answer basic questions correctly during the warm‐up phase (see details in the Supporting Information). Recruitment procedures, recruitment pool characteristics and inclusion criteria were the same as in Study 1. Sample sizes were set by conducting an a priori power analysis with G*power (v.3.1; Faul et al. 2007), assuming an effect size at the lower bound of the ones observed in Studies 1's generalization phase (*d* = 0.3). These analyses revealed that a minimum sample size of 96 participants was sufficient to achieve a power higher than 0.80 (*α* = 0.05) for comparing data against chance with a two‐tailed one sample Wilcoxon test. Following our recruitment efforts, more participants met our criteria in the schools contacted for the study and were included beyond the minimum of 96 participants.

#### 3.1.2 Apparatus

The apparatus was the same as in Study 1, except that stimuli were displayed using PsychoPy v2022.2.5 (Peirce et al. 2019).

#### 3.1.3 Stimuli

The full set of videos and audio used in this study is available at: https://osf.io/g5u79/?view_only=ddbd284bd1e348cdada3a7dd3ef90206.

#### 3.1.4 Procedure

**FIGURE 4:**
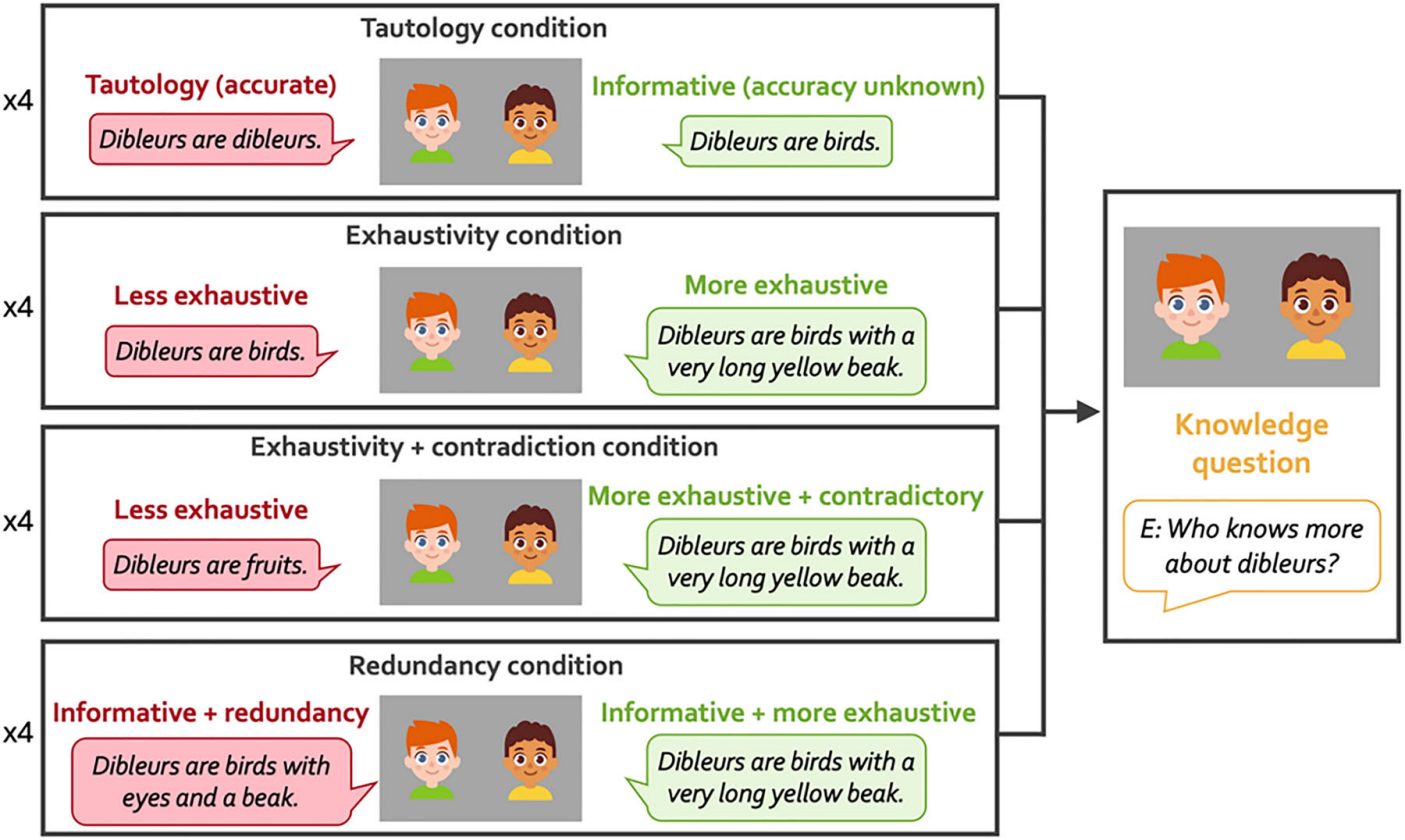
Trial sequence for the test phase of Study 2, per condition. *Note*: The text and speech bubbles shown in the figure serve illustration purposes. They did not appear in the videos shown to the participants. E = experimenter.

After a brief warm‐up phase (detailed in the Supporting Information), Study 2's participants were enrolled in 16 test trials in which pairs of characters (represented by drawings of children) provided information about the meaning of a novel label. On each trial, the children had to decide which character was more knowledgeable (Figure 4).

Study 2's participants were enrolled in four within‐subject conditions, with a block of four consecutive trials per condition. For all conditions, at the start of a trial, the experimenter said: *“Now, the two children will tell us about the [a novel label in the plural form, e.g., dibleurs]. Are you ready? Here we go!”* Then, the experimenter launched a video in which each of the two characters provided information about the unfamiliar label. What the characters said varied systematically across conditions, as follows.

##### 3.1.4.1 Tautology Condition

The tautology condition was based on Study 1. One character provided a tautology (e.g., *“Dibleurs are dibleurs.”)*, the other an informative claim (e.g., “*Dibleurs are birds.”*).

##### 3.1.4.2 Exhaustivity Condition

In the exhaustivity condition, one character provided a simple informative claim (e.g., “*Dibleurs are birds.”*), the other, a compatible but more exhaustive one (*“Dibleurs are birds with a very long yellow beak.”*).

##### 3.1.4.3 Exhaustivity + Contradiction Condition

The exhaustivity + contradiction condition was identical to the exhaustivity condition, but the two characters gave contradictory answers. (e.g., *“Dibleurs are fruits” vs. “Dibleurs are birds with a very long yellow beak.”*).

##### 3.1.4.4 Redundancy Condition

In the redundancy condition, both characters provided informative claims. However, one added redundant details (e.g., *“Dibleurs are birds with eyes and a beak”*), while the other added additional informative details (e.g., *“Dibleurs are birds with a very long yellow beak.”*).

In all conditions, the experimenter asked a test question at the end of each trial, once both characters had talked: “This child says that [dibleurs] are [answer of the first character], and this one says that [dibleurs] are [answer of the second character]. According to you, who knows more about [dibleurs]?” Once the child had responded, the experimenter moved on to the next trial, till the whole experiment was completed. Participants did not receive any feedback on whether their answers were correct or incorrect. In Study 2, a different pair of characters and a different novel label were used in each trial (the full list of labels is detailed in the Supporting Information).

#### 3.1.5 Counterbalanced Factors

The factors counterbalanced for Study 2 are detailed in the Supporting Information.

#### 3.1.6 Coding

For each test question, participants received a score of 1 if they chose the most informative character, and 0 otherwise.

### 3.2 Results

**FIGURE 5:**
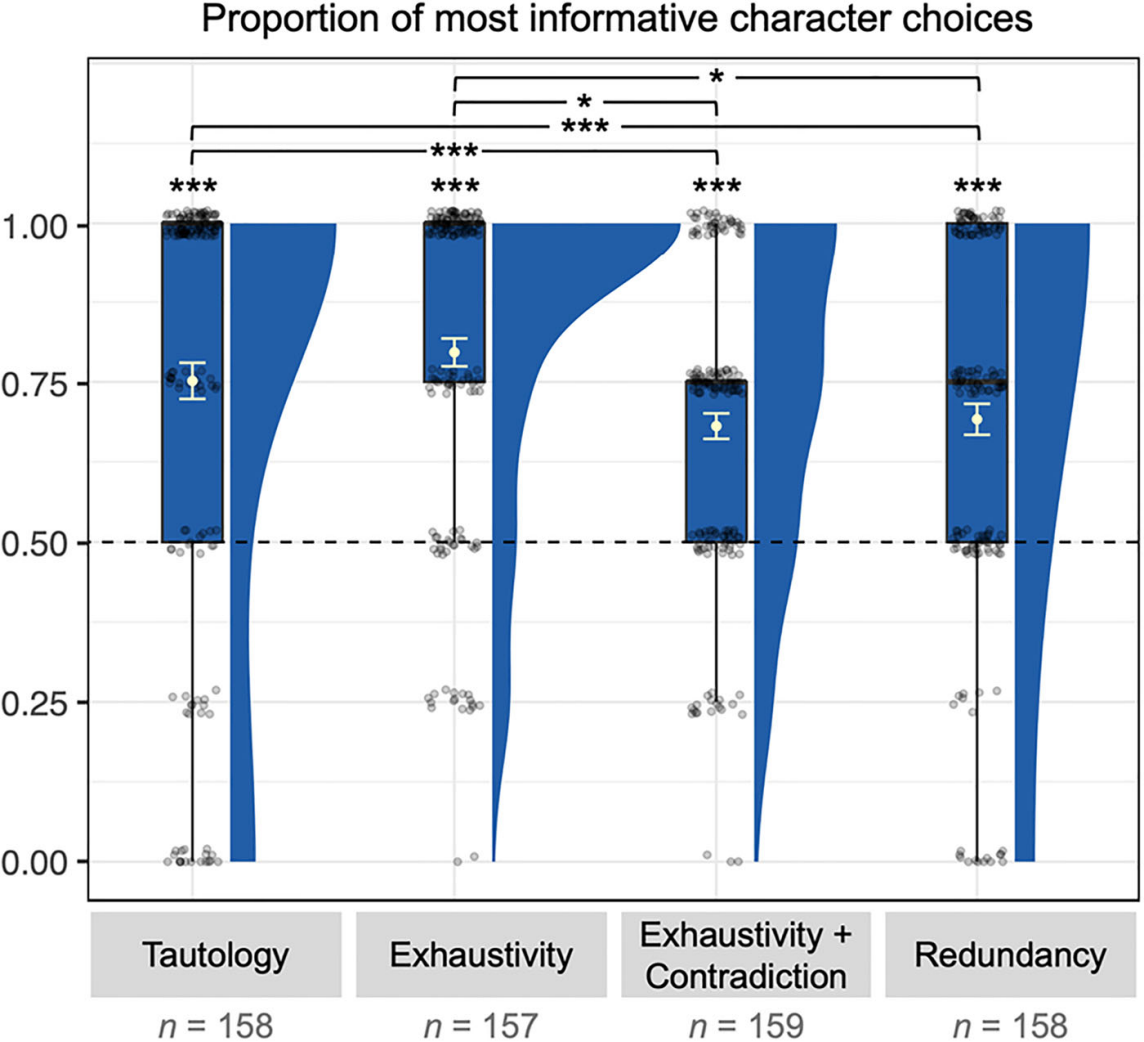
Proportions of choices of the most informative character in Study 2 per condition. *Note*: Yellow dots and error bars represent means and standard errors. Box plots represent data quartiles. Grey dots represent individual data points (with a slight jitter). Colored areas represent data density. Sample sizes vary slightly across conditions because a few children did not complete the full study. They are reported below each condition's name. Comparisons against chance level (0.5) by one‐sample Wilcoxon signed rank tests. Comparison across conditions by Tukey post‐hoc tests. Significance codes: *p* < 0.05 = *; *p* < 0.01 = **; *p* < 0.001 = ***.

As Figure 5 shows, when asked which character was more knowledgeable, children chose the most informative character significantly more often than predicted by chance in all conditions of Study 2 (all *p*s < 0.001, all *r*s > 0.54, one‐sample Wilcoxon tests, see detailed statistics in Table S6).

We also ran GLMMs with selected informant as a binary dependent variable (binomial distribution, logit link) on data from each condition. We constructed (1) a null model including gender, as well as the random effect of participants, and (2) a full model adding the fixed effects of condition and age (standardized) to the null model. Interactions between age and condition were not included in the full model as they were not statistically significant. In Study 2, the full model had a better fit than the null model (*χ* ^2^(4) = 52.89, *p* < 0.001, Likelihood Ratio Test).

As Figure 5 shows, Tukey post hoc tests indicated that Study 2's participants were more likely to select the most informative character in the tautology and in the exhaustivity conditions than in the other two conditions (tautology vs. exhaustivity + contradiction: *β* = 0.43, *SE* = 0.14, *z* = 3.11, *p_adj_* = 0.010; tautology vs. redundancy: *β* = 0.35, *SE* = 0.14, *z* = 2.58, *p_adj_* = 0.049; exhaustivity vs. exhaustivity + contradiction: *β* = 0.71, *SE* = 0.14, *z* = 5.03, *p_adj_* < 0.001; exhaustivity vs. redundancy: *β* = 0.64, *SE* = 0.14, *z* = 4.51, *p_adj_* < 0.001, Tukey adjusted *p* values for multiple comparisons). Post‐hoc contrasts revealed no other statistically significant results.

**FIGURE 6:**
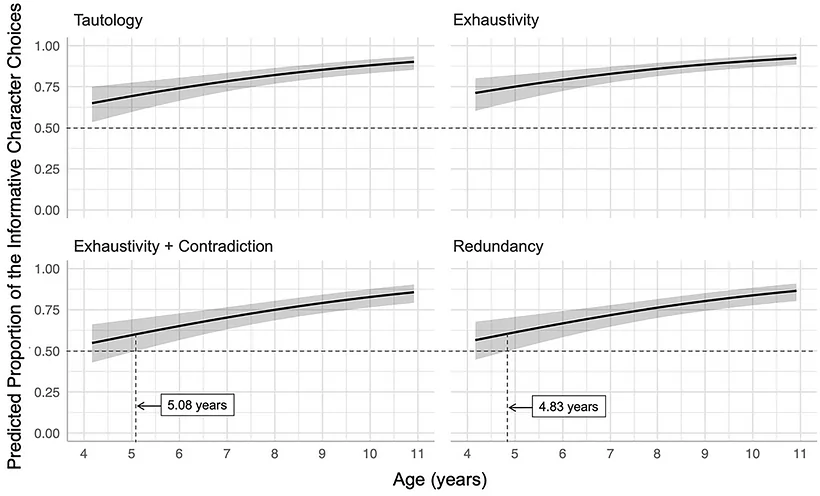
Predicted proportions of choices of the informative character in Study 2 per condition. *Note*: Shaded areas represent 95% confidence intervals. Vertical black dotted lines represent the age at which confidence interval did not include chance (0.5), represented by horizontal dotted black lines. Predictions reported on the figure cover the full age range of participants in Study 2 (from 49 to 131 months). For ease of reading, standardized age values used to build the model were back‐transformed to their original values.

Estimates based on the full model indicated that participants selected the most informative informant more often than predicted chance across the entire age range in the tautology and exhaustivity conditions (Figure 6, top part). In the exhaustivity + contradiction and redundancy conditions, this above‐chance performance emerged by ages 5.08 and 4.83 years, respectively (Figure 6, bottom part).

## 4 General Discussion

Children routinely encounter new claims in areas they know little about, such as the fact that the universe is 13.8 billion years old, that chlorophyll makes plants green, or that writing emerged independently in Mesopotamia, China, and Mesoamerica. Even when they have no information about the accuracy of these statements or about the people making them, children nevertheless rely on systematic strategies to assess the reliability of informants. They seek out and endorse informative statements that might contribute to their learning. They also build trust in informants who provide such claims, using speakers’ informativeness as a cue to their knowledge.

### 4.1 Children's Reliance on Claims and Individuals That Are Informative

In Study 1, children relied more on informative statements whose accuracy was unknown to them than on uninformative tautologies which were necessarily accurate. Later, when they needed to learn the names of new objects, children preferred to seek and endorse the testimony of non‐tautological individuals who had been informative in the past—even if their accuracy had never been established—rather than from tautological informants who demonstrated accuracy but provided no information. By age five, this preference for relying on informative testimonies and informative speakers was significant across all experimental measurements.

Thus, Study 1 suggests that children are not fully conservative from an epistemic standpoint. They value informativeness in testimonies and in informants, even without direct evidence about the accuracy of the information provided. This risk‐taking strategy is needed to make it possible for them to learn in unfamiliar domains, where informants might provide them with information whose accuracy they cannot yet assess.

However, these risks remained regulated. In the control condition, the non‐tautological informant proved to be inaccurate, by contradicting the children's pre‐established knowledge. In that case, the children shifted their preference toward the tautological informant as early as age four, even if the latter was completely uninformative. Children therefore do not avoid uninformative sources at all costs. Even in the context of our study, they remained sensitive to information about individuals’ accuracy and they adjusted their trust accordingly.

When learning individually, children actively search for evidence that might help them increase their knowledge and reduce their uncertainty (Kidd and Hayden 2015; Poli et al. 2024; Schulz 2012). This process is guided by children's early emerging sensitivity to properties of evidence that go beyond accuracy, such as the amount of information conveyed by stimuli (Aguirre et al. 2022). Our findings indicate that when evaluating informants, children also do not just consider the accuracy of communicated contents, but also their informativeness. Previous research had shown that children evaluate negatively informants who, by being under‐informative, imply something inaccurate (Bass et al. 2022; Gweon et al. 2014; Gweon and Asaba 2018; Katsos and Bishop 2011). We find that even when under‐informative individuals are not inaccurate, children nonetheless trust them less than informative ones.

### 4.2 The Quantity‐Competence Presumption

Study 2 investigated the mechanisms underlying children's preference for learning from informative individuals. It tested whether children apply the quantity‐competence presumption: all else being equal, a more informative individual implies that she knows more. In line with our hypothesis, children attributed less knowledge to tautological and less exhaustive speakers than to more informative ones by age four. By age five, children followed the quantity‐competence presumption when presented with even subtler contrasts. They still expected the more informative individual to know more when a less and a more exhaustive speaker contradicted each other. They also did so when the less informative speaker offered redundant information that did not involve explicit repetitions. Importantly, children's inferences tracked graded degrees of informativeness. They judged speakers of tautologies (*a dibleur is a dibleur*) as less knowledgeable than speakers of general claims (*a dibleur is a bird*), who in turn were judged less knowledgeable than speakers of more specific elaborations (*a dibleur is a bird with a very long yellow beak*). Consequently, children assess the informativeness of testimony in degrees, and treat it as a graded signal of knowledge.

This result has consequences for the study of theory of mind, specifically, children's capacity to represent degrees of knowledge in others. Research on children's attribution of knowledge and ignorance to others typically focuses on binary conceptions of knowledge—for example, whether someone has seen something or not (e.g., Aguirre et al. 2022; Bernard et al. 2025; Luo and Baillargeon 2007; Phillips et al. 2021; Schuwerk et al. 2021). This view of knowledge is not graded: it supports monitoring whether someone is aware (or not) of something, not whether someone knows more or less about a topic. Here, we find that children monitor the amount of knowledge someone has about a topic in a graded fashion. This result opens perspectives for studying the early development of representations of amounts of knowledge.

Additionally, Study 2's results must be understood in relation to prior work on children's sensitivity to circular explanations. Mills et al. (2017) showed that when evaluating a single explanation in isolation, even 6‐year‐olds sometimes struggle to reliably detect tautological explanations as weaker than noncircular ones; by contrast, they perform better when explanations are presented in pairs contrasting circular explanations with a more informative one. The present paradigm likewise relies on a contrastive design, and our findings should therefore not be taken as evidence that children evaluate informativeness in absolute terms, without a contrasting utterance. That said, the contrastive structure used in Study 2 mirrors a frequent feature of real learning environments, where children often encounter multiple sources providing competing testimony.

Because the quantity‐competence presumption is intuitive and observed very early on, it is also susceptible to exploitation. A highly informative speaker can elicit an unwarranted impression of competence. The latter can be exploited for deceptive purposes or might distort learners’ assessment of competence. For instance, large language models (LLMs) can quickly generate informative responses in many domains. If listeners intuitively treat informativeness as an indicator of knowledge, such systems can trigger impressions of high competence even before their accuracy is checked—thereby contributing to the perception of LLMs as super‐informants. Conversely, high‐informativeness conveyed through excessive precision carries reputational risks. It may suggest overconfidence or dishonesty by implying an implausible degree of competence. In fact, adults tend to dismiss advice that is implausibly precise (Schultze and Loschelder 2021). Furthermore, when a highly informative speaker is later found to be wrong, the resulting reputational damage might be amplified—in such cases, the error contradicts an implicit claim of high competence. Whether children exhibit similar backlash effects toward highly informative speakers whose contribution is later found to be inaccurate is an important topic for future research.

Importantly, more information is not always good. As early as age five, children react to overinformativeness (Morisseau et al. 2013). They also prefer to learn from teachers who provided just the right amount of information than from those who exhaustively restated what learners already knew (Gweon et al. 2018). Detecting over‐informativeness is not trivial, however. It requires a sufficient level of prior knowledge of the subject matter, and about what the audience already knows—making it possible to distinguish relevant from superfluous information. Our paradigm deliberately placed children in a state of near‐total ignorance—they knew nothing about the entities described. Such contexts, frequently encountered by young children, require trusting teachers to select the relevant information worth knowing. Thus, the upper limit of the quantity‐competence presumption likely depends on the learners’ level of knowledge.

### 4.3 Limits and Future Research Directions

Our study has several limitations, some of which are noteworthy and open perspectives for future research. First, in Study 2, children's inferences about informants’ knowledge could not be explained solely by the length of the utterances, as this factor was controlled for in the tautology and redundancy conditions. Yet, the nature of the underlying mechanisms supporting these inferences remains an open question: it may range from simple heuristics—such as a direct association between “more information” and “more knowledge”—to more explicit reasoning, allowing children to process that a speaker who spontaneously provides detailed information offers indirect evidence of their knowledge. A second related, largely open question is whether children represent the epistemic grounds that make the quantity‐competence presumption reliable—for example, the fact that detailed and coherent testimony requires domain knowledge that uninformed speakers generally lack, or that making more specific claims entails greater epistemic responsibility. The question of whether children explicitly represent these foundations, implicitly rely on them, or simply react to surface informativeness without representing its epistemic basis constitutes an important avenue of future research.

Third, we found that children infer a speaker's level of knowledge based on the amount of information they provide. Yet, a speaker's level of informativeness may stem from many different causes beyond their knowledge. Informants might deliberately withhold information or fail to accurately determine what their audience needs to know. The question of whether and how children distinguish among these possibilities is therefore an important area of future research.

## Author Contributions

**Cyann Bernard**: conceptualization, investigation, writing – original draft, methodology, visualization, writing – review and editing, software, formal analysis, data curation, validation. **Adeline Depierreux**: investigation, writing – review and editing. **Olivier Mascaro**: conceptualization, funding acquisition, writing – original draft, methodology, visualization, writing – review and editing, software, formal analysis, project administration, supervision, resources.

## Ethics Statement

The research reported in this manuscript followed the guidelines of the Declaration of Helsinki and was approved by an independent ethics committee (CER U‐Paris Cité, IRB: 00012023‐102). The parents of all participants gave their informed written consent before their inclusion in any of the studies.

## Conflicts of Interest

The authors declare no conflicts of interest.

## Supporting information




**Supporting Information**: desc70276‐supp‐0001‐SuppMat.docx

## Data Availability

The data that support the findings of this study are openly available in the Open Science Framework website at the following links: https://osf.io/cfm5z/files/osfstorage?view_only=2b80af72d73045c4bd3b5bb6971e8ed2 and https://osf.io/g5u79/files/osfstorage?view_only=ddbd284bd1e348cdada3a7dd3ef90206.
