# Supplementary material for: The Quantity‐Competence Presumption: Children Use Informativeness to Calibrate Trust and Infer Speakers' Knowledge
Source: Dev Sci. 2026 Aug 25;29(5):e70276. doi: 10.1111/desc.70276 (PMC13505260; doi:10.1111/desc.70276)
Supplement: Supplementary file 1 [file DESC-29-e70276-s001.docx]

**Supporting information for**

**The Quantity-Competence Presumption:**

**Children Use Informativeness to Calibrate Trust and Infer Speakers' Knowledge**

**This file contains the following sections**

**1. Recruitment Pool Characteristics and Inclusion criteria**

**2. Deviations from Pre-registration**

**2.1. Study 1**

**2.2 All Studies**

**3. Additional information about Study 1**

**3.1 Additional Procedural Details**

**3.2 Stimuli**

**Table S1 —** *Set of Labels Used in the Initial Phase of Study 1.*

**Table S2 —** *Pseudo-words Used to Name Unfamiliar Objects in the Generalization Phase of Study 1_._*

**3.3 Detailed Statistics**

**Table S3 —** *Average and Median Proportions of Choices of the Non-tautological Character (Ask Questions), or of her Testimony (Endorse Questions) and Comparisons Against Chance Level, per Condition, Phase and Question Type in Study 1.*

**Table S4 —** *Average and Median Proportions of Choices of the Non-tautological Character (Ask Questions), or of her Testimony (Endorse Questions) and Comparisons Across Condition (test vs. control) per Question Type in Study 1.*

**3.4 Additional Analyses: Learning Effects During the Initial Phase**

**4. Additional information about Study 2**

**4.1 Additional Procedural Details**

**4.2 Stimuli**

**Table S5 —** *Sentences Used in Study 2*

**4.3 Detailed Statistics**

**Table S6 —** *Average and Median Proportion of Choices of the Most Informative Character in Study 2 per Condition and Comparisons Against Chance Level.*

**1. Recruitment Pool Characteristics and Inclusion criteria**

The participants were recruited in a large French urban area (Paris and its surroundings), by contacting schools and sending e-mails to the families who volunteered to be registered in the database of the infancy research center. Children (and their families) did not receive any financial incentive to enroll in our study. Each participating child received instead a “little scientist diploma” as a thank you gesture. Each child participated in only one of the studies reported in this paper. To be included, participants needed to speak French and have no known or suspected developmental disorder. Data collection happened in 2024-2025, from Monday to Saturday, between 8:30 and 5:30 pm.

This section also reports official data about the living conditions the recruitment area for all Studies (extracted in August 2025 from the repository of the National Institute of Statistics and Economics Studies, <https://www.insee.fr/fr/statistiques/4481962#titre-bloc-11> <https://www.insee.fr/fr/statistiques/8327885> and <https://www.insee.fr/fr/statistiques/7681747?sommaire=7679565>). To make the characterization of the recruitment area more informative, French national averages are also reported. In the recruitment area, the median annual income per household is slightly above the national average (Recruitment area: €25,210; French average: €23,000). In the recruitment area, about half of the out-of-school population has a college degree or equivalent (Recruitment area: 45.2%; French average: 32.6%). The poverty and unemployment rates of the recruitment area are respectively 15.5% and 8.5%; they are roughly comparable to the national averages (French average poverty rate: 14.4%; French average unemployment rate: 8.8%). In the recruitment area, 69% of children live in a so-called "traditional" family, where all the children living in the home are those of the couple (French national average: 68%). Twenty-two percent of children live in a single-parent family (French average number of single parent families: 21%). In short, in comparison to the national average, the population in the recruitment area is more educated and slightly more affluent. Data collection occurred in France, where collecting information on race/ethnicity is forbidden.

**2. Deviations from Pre-registration**

2.1 **Study 1**

Our data did not fulfill the requirements for parametric analyses in any of the Studies. Thus, data were not analyzed with t-tests, unlike what was pre-registered for Study 1. Instead, we used Wilcoxon and Mann-Whitney U tests. For consistency, we report power analyses for Wilcoxon tests when justifying sample sizes for Study 1.

We excluded data from the two first trials of the initial phase when analyzing data from the ask question. This choice was made to focus specifically on trials in which participants had received enough evidence about the behaviors of the two characters, and of their consistency, when choosing from whom to ask information.

An exploratory analysis was added to assess learning effects during the initial phase of Study 1. This exploratory analysis compared children’s behaviors on the very first trial of the initial phase to chance. Its purpose was to assess whether participants would still endorse the most informative, or most accurate testimony, while having little information about the characters (see details below).

**2.2 All Studies**

For all Studies, age was standardized (not just mean-centered) when building Generalized Linear Mixed Models. For all Studies, in addition to the analyses reported in the paper, we also pre-registered a set of analyses on the data divided into three age groups. The purpose of these analyses was to determine at which age the pattern of results observed for the entire set of participants would become significant. We did not implement this set of analyses because estimates based of pre-registered GLMMs already provided us with a more precise alternative to address this question, by pin-pointing the exact age from which patterns of significant results emerged.

**3. Additional information about Study 1**

**3.1 Additional Procedural Details**

***Warm-up Phase.*** At the very beginning of Study 1, the experimenter explained the study to the participants. First, she showed a still image of a rabbit and a cow hand-puppets, each placed on one side of the screen. The experimenter said: “*Look, we are going to play with these two puppets. There is a rabbit and a cow”*. Next, the experimenter asked control general knowledge questions: *“Can you please show me the rabbit? And the cow? Now, the rabbit and the cow will introduce themselves to us. Are you ready? Let’s go!”* Participants that failed on the control general knowledge questions were excluded from analysis. Next, the participants were shown a short video of the two characters greeting them and saying “*Hi, I am the rabbit! Hi, I am the cow!*”.

***Counterbalanced Factors.*** Trials were presented in a fixed order (see Tables S1 and S2 in the Supporting information). For each participant and each phase, the identity of the character speaking first (tautological vs. non-tautological) was counterbalanced across trials. When asking the endorse questions, the experimenter repeated what the characters said in the order in which they had spoken during the trial (starting with the character who spoke first). Additionally, the following factors were counterbalanced across participants: the identity of the character speaking first, the screen side occupied by each character, the assignment of roles (tautological vs. non-tautological) to animal characters, and for each given novel label, which character uttered it during the generalization phase.

**3.2 Stimuli**

**Table S1**

*Set of Labels Used in the Initial Phase of Study 1.*

| Trial number | Pseudo-words used by both informants in the test condition | Familiar words used by both informants in the control condition | Words used by the non-tautological character to explain what the pseudo-word or the familiar word is |
| --- | --- | --- | --- |
| 1 | Dibleur | Shoe (“*Chaussure*”) | Bird (“*Oiseau*”) |
| 2 | Granée | Car (“*Voiture*”) | Fruit (“*Fruit*”) |
| 3 | Mouval | Book (“*Livre*”) | Cake (“*Gâteau*”) |
| 4 | Ludine | Dog (“*Chien*”) | Flower (“*Fleur*”) |

*Note*. The French words used in the experiment are reported within parentheses.

**Table S2**

*Pseudo-words Used to Name Unfamiliar Objects in the Generalization Phase of Study 1_._*

| Trial number | Pseudo-words used by one of the informants | Pseudo-words used by the other informant |
| --- | --- | --- |
| 1 | Tralet | Soileau |
| 2 | Pujette | Gafence |
| 3 | Cuidor | Vamont |
| 4 | Molée | Bossaire |

*Note*. Whether each given novel label was uttered by the informative or by the uninformative character was counterbalanced across participants.

**3.3 Detailed Statistics**

**Table S3**

*Average and Median Proportions of Choices of the Non-tautological Character (Ask Questions), or of her Testimony (Endorse Questions) and Comparisons Against Chance Level, per Condition, Phase and Question Type in Study 1.*

| Condition | Phase | Question | Descriptive statistics | | | | | One sample Wilcoxon test | | |
| --- | --- | --- | --- | --- | --- | --- | --- | --- | --- | --- |
|  |  |  | *n* | *M* | *Mdn* | *SD* | *W_+_* | | *p* | r [95% CI] |
| Test | Initial | Ask | **115** | **.62** | **.50** | **.32** | **1127.5** | | **<.001** | **0.36 [0.18, 0.5]** |
|  |  | Endorse | **115** | **.73** | **.75** | **.29** | **3731** | | **<.001** | **0.64 [0.51, 0.75]** |
|  | Generalization | Ask | **115** | **.69** | **.75** | **.28** | **3041.5** | | **<.001** | **0.56 [0.41, 0.69]** |
|  |  | Endorse | **115** | **.59** | **.50** | **.30** | **2012.5** | | **.001** | **0.29 [0.12, 0.45]** |
| Control | Initial | Ask | **110** | **.33** | **.50** | **.31** | **252** | | **<.001** | **0.48 [0.32, 0.62]** |
|  |  | Endorse | **110** | **.11** | **.00** | **.20** | **7** | | **<.001** | **0.88 [0.85, 0.92]** |
|  | Generalization | Ask | **110** | **.23** | **.25** | **.26** | **161** | | **<.001** | **0.72 [0.62, 0.80]** |
|  |  | Endorse | **110** | **.32** | **.25** | **.29** | **563** | | **<.001** | **0.51 [0.35, 0.63]** |

*Note.* Comparisons against the proportion expected by chance by two-tailed one-sample Wilcoxon tests. Statistically significant results in bold.

**Table S4**

*Average and Median Proportions of Choices of the Non-tautological Character (Ask Questions), or of her Testimony (Endorse Questions) and Comparisons Across Conditions (test vs. control) per Question Type in Study 1.*

| Phase | Question | Test condition | | | Control condition | | | Mann-Whitney U test | | |
| --- | --- | --- | --- | --- | --- | --- | --- | --- | --- | --- |
|  |  | *n* | *M* | *Mdn* | *n* | *M* | *Mdn* | *U* | *p* | r [95% CI] |
| Initial | Ask | **115** | **.62** | **.50** | **110** | **.33** | **.50** | **9108** | **<.001** | **0.42 [0.30, 0.52]** |
|  | Endorse | **115** | **.74** | **.75** | **110** | **.11** | **.00** | **11888** | **<.001** | **0.79 [0.73, 0.84]** |
| Generalization | Ask | **115** | **.69** | **.75** | **110** | **.23** | **.25** | **10946** | **<.001** | **0.65 [0.57, 0.73]** |
|  | Endorse | **115** | **.59** | **.50** | **110** | **.33** | **.25** | **9228** | **<.001** | **0.41 [0.29, 0.51]** |

*Note.* Comparisons across conditions (test vs. control) by two-tailed Mann-Whitney U tests. Statistically significant results in bold.

**3.4 Additional Analyses: Learning Effects During the Initial Phase**

Additional complementary analyses assessed learning effects during the initial phase. These analyses were designed to evaluate whether participants would gradually become less likely to rely on the uninformative character in Study 1’ test condition, and on the non-tautological character in Study 1’control condition.

For these analyses, we ran GLMMs with asking or endorsing information from the non-tautological character for the ask and endorse questions as a binary dependent variable (binomial distribution, logit link) on data from the initial phase (trials 1 to 4). We built (1) an initial model including the fixed effects of age (standardized), gender, and Study, as well as the random effect of participants, and (2) a trial number model adding the fixed effect of Trial number (1, 2, 3 or 4) to the initial model. We included interactions between trial number and age, and interactions between trial number and study in the full model if they were statistically significant. They were dropped from the full model otherwise.

These analyses were run twice, each time with answers on one of the questions (ask or endorse) during the initial phase as the repeated dependent measure. For the analyses ran on answers to the ask question, the trial number model provided a better fit than the initial model (*χ*^2^(2) = 9.95, *p* = .007, Likelihood Ratio Test). Moreover, the trial number model revealed an interaction between condition and trial number (*β* = –.37, *z* = –3.04, *p* = .002). Thus, the effect of trial number on children’s answer to the ask question varied across conditions (with participants becoming more likely to ask help from the non-tautological character across trials in the test condition, and showing the opposite pattern in the control condition). For the analyses ran on answers to the ask question, the full model revealed no other significant effect.

For the analyses ran on answers to the endorse question, the trial number model did not provide a better fit than the initial model (*χ*^2^(2) = 1.65, *p* = .439, Likelihood Ratio Test) Moreover, the trial number model revealed no main effect of trial number, and no interaction between condition and trial number. In sum, when asked to endorse the testimony of one of the informants, participants did not change their behaviors as they gained more information about the character during the initial phase. Thus, during the initial phase of Study 1, children presumably relied on the information contained within each testimony (rather than on its source) to determine whether to endorse it.

To confirm this interpretation, we ran an exploratory (unplanned) analysis of participants’ answers to the very first endorse question. At that time, participants had received no other piece of information about characters than their initial testimony. On the first trial of the initial phase, participants enrolled in the control condition of Study 1 already endorsed the testimony from the non-tautological character more often than predicted by chance (89 participants out of 115, *p* < .001, *g* = .27, 95% CI = [.19, .35], two choice binomial test). In the control condition, participants showed the opposite pattern in the first trial of the initial phase (18 participants out 110 endorsed the testimony of the non-tautological — but inaccurate— character, *p* < .001, *g* = –.34, 95% CI = [–.40, –.25], two choice binomial test).

In short, these analyses indicated that during the initial phase, children’s tendency to ask the non-tautological character for help changed across trials, indicating that they used cumulative evidence about informants’ characteristics, gathered across trials, when deciding whom to ask for help. In contrast, endorsement patterns did not change trial-by-trial during the initial phase, and they differed from chance from the very first trial. Thus, during the initial phase, children appeared to use the content of the characters’ testimony (rather than evidence about characters’ reliability accumulating across trials) to determine whether to endorse it.

**4. Additional information about Study 2**

**4.1 Additional Procedural Details**

***Warm-up Phase.*** At the very beginning of Study 2, the experimenter explained the study to the participants. She showed a still image with two drawings representing children (one on the left and one on the right of the screen), and said: “*Alright, in this game, you will see children* *talking about lots of different objects. Two at a time, the children will each say something about* *an object, and you will tell me each time which one knows the object better, okay? To start, we* *will do two practice rounds to practice, ok?”*

Next, participants were enrolled in two warm-up trials in which characters were asked about familiar categories (carrots and elephants). At the beginning of each warm-up trial, the experimenter said: *“Now, the two children will tell us* *about [name of the familiar category used in the trial, e.g., carrots]. Let’s ask them what [carrots] are. Are* *you ready? Here we go!”* Then, the experimenter launched a video in which one informant made an accurate claim (e.g., *“Carrots are vegetables.”*), while the other expressed ignorance (*“I don’t know.”*). Next, the experimenter asked a warm-up question to the child: *“This child says that [carrots] are [vegetables], and this one says they don't know what [carrots] are. According to you,* *who knows more about [carrots]?”* While asking questions, the experimenter referred to the characters and to the content of their answers in the order they spoke in the video used in the trial. She pointed to each informant while mentioning them (i.e., when saying “this child”, and “this one”). Participants that did not select the character who made an accurate claim in any of the two warm-up trials were excluded from the analysis.

For warm-up trials, the following factors were randomized across participants: the order of the two trials (carrots first vs. elephants first); the side of the character who claimed to be ignorant (left vs. right side of the screen); the order in which characters spoke (ignorant first vs. knowledgeable first); and the order of the videos presenting the pairs of characters (two girls first vs. two boys first).

***Counterbalanced Factors.*** Condition order was randomized. We counterbalanced across participants: which sentence sets and which novel labels were used in each condition (Table S5), which character uttered the most informative sentence, and characters' genders (alternating across trials, with characters having the same gender in each given trial). In addition, we counterbalanced the following factors across trials, for each participant and each condition: the side of the screen occupied by the most informative character (left or right side of the screen; the position of the least informative character varied accordingly); which character spoke first (most informative first vs. least informative first); and which character the experimenter referred to first while asking test questions (most informative first vs. least informative first).

**4.2 Stimuli**

***Full List of Novel Labels.*** The novel labels used in Study 2 were the following: *dibleurs, molées, mireuses, pujettes, tralets, cuidors, castelles, bossaires, ludines, vamonts, tavalies, gafences, granées, soileaux, mouvals, rémants*.

**Table S5**

*Sentences Used in Study 2*

|  | Conditions for which the sentences were used | | | | |
| --- | --- | --- | --- | --- | --- |
|  | Tautology | Tautology  Exhaustivity | Exhausitivity  Exhaustivity + Contradiction  Redundancy | Redundancy | Exhaustivity + Contradiction |
|  | Sentences | | | | |
| G  R  O  U  P  A | Les dibleurs sont des dibleurs.  Dibleurs are dibleurs. | Les dibleurs sont des oiseaux.  Dibleurs are birds. | Les dibleurs sont des oiseaux avec un très long bec jaune.  Dibleurs are birds with a very long yellow beak. | Les dibleurs sont des oiseaux avec des yeux et un bec.  Dibleurs are birds with eyes and a beak. | Les dibleurs sont des fruits.  Dibleurs are fruits. |
|  | Les molées sont des molées.  Molées are molees. | Les molées sont des voitures.  Molées are cars. | Les molées sont des voitures hautes et très puissantes.  Molées are tall and very powerful cars. | Les molées sont des voitures qui ont quatre roues.  Molées are cars with four wheels. | Les molées sont des chiens.  Molées are dogs. |
|  | Les mireuses sont des mireuses.  Mireuses are mireuses. | Les mireuses sont des pinceaux.  Mireuses are paintbrushes. | Les mireuses sont des pinceaux ronds et très larges.  Mireuses are round and very wide paintbrushes. | Les mireuses sont des pinceaux qui servent à peindre.  Mireuses are paintbrushes used for painting. | Les mireuses sont des livres.  Mireuses are books. |
|  | Les pujettes sont des pujettes.  Pujettes are pujettes. | Les pujettes sont des bonbons.  Pujettes are candies. | Les pujettes sont des bonbons durs aux fruits.  Pujettes are hard fruit candies. | Les pujettes sont des bonbons qui se mangent.  Pujettes are candies that are eaten. | Les pujettes sont des fleurs.  Pujettes are flowers. |
| G  R  O  U  P  B | Les tralets sont des tralets.  Tralets are tralets. | Les tralets sont des arbres.  Tralets are trees. | Les tralets sont des arbres très grands avec des fleurs blanches.  Tralets are very large trees with white flowers. | Les tralets sont des arbres avec un tronc et des branches.  Tralets are trees with a trunk and branches. | Les tralets sont des chiens.  Tralets are dogs. |
|  | Les cuidors sont des cuidors.  Cuidors are cuidors. | Les cuidors sont des fromages.  Cuidors are cheeses. | Les cuidors sont des fromages ronds au lait de chèvre.  Cuidors are round goat's milk cheeses. | Les cuidors sont des fromages que l’on peut manger.  Cuidors are cheeses that can be eaten. | Les cuidors sont des fruits.  Cuidors are fruits. |
|  | Les castelles sont des castelles.  Castelles are castelles. | Les castelles sont des jouets.  Castelles are toys. | Les castelles sont des jouets qui font de la musique.  Castelles are toys that make music. | Les castelles sont des jouets qui servent à jouer.  Castelles are toys used for playing. | Les castelles sont des fleurs.  Castelles are flowers. |
|  | Les bossaires sont des bossaires.  Bossaires are bossaires. | Les bossaires sont des poissons.  Bossaires are fish. | Les bossaires sont des poissons noirs qui mangent des algues.  Bossaires are black fish that eat algae. | Les bossaires sont des poissons qui vivent dans de l’eau.  Bossaires are fish that live in water. | Les bossaires sont des livres.  Bossaires are books. |
| G  R  O  U  P  C | Les ludines sont des ludines.  Ludines are ludines. | Les ludines sont des bateaux.  Ludines are boats. | Les ludines sont des bateaux qui vont très vite.  Ludines are boats that go very fast. | Les ludines sont des bateaux qui vont sur l’eau.  Ludines are boats that sail on water. | Les ludines sont des fleurs.  Ludines are flowers. |
|  | Les vamonts sont des vamonts.  Vamonts are vamonts. | Les vamonts sont des plats.  Vamonts are dishes. | Les vamonts sont des plats de riz et d’oignons.  Vamonts are rice and onions dishes. | Les vamonts sont des plats que l’on peut manger.  Vamonts are dishes that can be eaten. | Les vamonts sont des chiens.  Vamonts are dogs. |
|  | Les tavalies sont des tavalies.  Tavalies are tavalies. | Les tavalies sont des chaussures.  Tavalies are shoes. | Les tavalies sont des chaussures qui sont bleues et noires.  Tavalies are shoes that are blue and black. | Les tavalies sont des chaussures qui se portent aux pieds.  Tavalies are shoes that are worn on the feet. | Les tavalies sont des livres.  Tavalies are books. |
|  | Les gafences sont des gafences.  Gafences are gafences. | Les gafences sont des chapeaux.  Gafences are hats. | Les gafences sont des chapeaux qui ont des plumes toutes douces.  Gafences are hats with soft feathers. | Les gafences sont des chapeaux qui se portent sur la tête.  Gafences are hats that are worn on the head. | Les gafences sont des fruits.  Gafences are fruits. |
| G  R  O  U  P  D | Les granées sont des granées.  Granées are granées. | Les granées sont des machines.  Granées are machines. | Les granées sont des machines qui creusent des trous dans la terre.  Granées are machines that dig holes in the ground. | Les granées sont des machines qui servent à faire des choses.  Granées are machines that are used to doing things. | Les granées sont des fleurs.  Granées are flowers. |
|  | Les soileaux sont des soileaux.  Soileaux are soileaux. | Les soileaux sont des ciseaux.  Soileaux are scissors. | Les soileaux sont des ciseaux qui coupent le tissu.  Soileaux are scissors that cut fabric. | Les soileaux sont des des ciseaux qui servent à couper.  Soileaux are scissors that are used to cut. | Les soileaux sont des fruits.  Soileaux are fruits. |
|  | Les mouvals sont des mouvals.  Mouvals are mouvals. | Les mouvals sont des gâteaux.  Mouvals are cakes. | Les mouvals sont des gâteaux très sucrés aux fraises.  Mouvals are very sweet strawberry cakes. | Les mouvals sont des gâteaux que l’on peut manger.  Mouvals are cakes that can be eaten. | Les mouvals sont des chiens.  Mouvals are dogs. |
|  | Les rémants sont des rémants.  Rémants are rémants. | Les rémants sont des chevaux.  Rémants are horses. | Les rémants sont des chevaux marron clair avec des taches blanches.  Rémants are light brown horses with white spots. | Les rémants sont des chevaux avec une tête et quatre pattes.  Rémants are horses with a head and four legs. | Les rémants sont des livres.  Rémants are books. |

*Note.* In black: original sentences in French; in grey: English translations. The lengths of the two informants’ sentences are equated in the Tautology and Redundancy conditions (same number of syllables and words). Four groups of sentences were used (A, B, C and D). Each child was enrolled in all four conditions (using one different group of sentences per condition). Across all children, all groups of sentences and all labels were used equally often for each of the conditions.

**4.3 Detailed Statistics**

**Table S6**

*Average and Median Proportion of Choices of the Most Informative Character in Study 2 per Condition and Comparisons Against Chance Level.*

| Condition | Descriptive statistics | | | | One sample Wilcoxon test | | |
| --- | --- | --- | --- | --- | --- | --- | --- |
|  | *n* | *M* | *Mdn* | *SD* | *W_+_* | *p* | r [95% CI] |
| Tautology | **158** | **.75** | **1.00** | **.36** | **8634** | **<.001** | **0.58 [0.45, 0.69]** |
| Exhaustivity | **157** | **.80** | **1.00** | **.27** | **8757** | **<.001** | **0.74 [0.66, 0.81]** |
| Exhaustivity + Contradiction | **159** | **.68** | **.75** | **.25** | **6280** | **<.001** | **0.59 [0.48, 0.69]** |
| Redundancy | **158** | **.69** | **.75** | **.30** | **5754** | **<.001** | **0.54 [0.4, 0.66]** |

*Note.* Sample sizes vary slightly across conditions because a few participants did not complete the full set of conditions. Comparisons against the proportion expected by chance by two-tailed one-sample Wilcoxon tests. Statistically significant results in bold.
